# Clinical significance and discriminatory performance of the serum KL-6 level as a biomarker for severe *Chlamydia pneumoniae* infection in children

**DOI:** 10.3389/fcimb.2026.1791097

**Published:** 2026-10-16

**Authors:** Lei Ding, Sufang Chen, Huafeng Song, Shuting Cheng, Meng Shao, Hongyan Zhang, Jun Hua, Fei Gao, Junchi Xu

**Affiliations:** 1 Department of Clinical Laboratory, Children’s Hospital of Wujiang District, Suzhou, China; 2 School of Humanities and Management, Youjiang Medical University for Nationalities, Baise, China; 3 Department of Clinical Laboratory, The Fifth People’s Hospital of Suzhou, Suzhou, China; 4 Department of Clinical Laboratory, The Affiliated Suzhou Hospital of Nanjing Medical University, Suzhou, China; 5 Department of Clinical Laboratory, Suzhou Municipal Hospital, Suzhou, China

**Keywords:** biomarker, *Chlamydia pneumoniae*, KL-6, pediatrics, pneumonia

## Abstract

**Objective:**

To characterize the expression and evaluate the clinical utility for assessing disease severity of the serum Krebs von den Lungen-6 (KL-6) level in children with mild and severe acute *Chlamydia pneumoniae* (*C. pneumoniae*) infection.

**Methods:**

A total of 68 hospitalized children with acute lower respiratory tract infection caused by *C. pneumoniae* (48 mild cases and 20 severe cases) who were admitted to Children’s Hospital of Wujiang District from May 2024 to April 2025 were enrolled, with 19 healthy children included in the control group. Serum KL-6 levels, *C. pneumoniae*-IgM levels, and routine laboratory indicators were measured. The correlation between the KL-6 level and clinical features was analyzed, and discriminatory performance of the KL-6 level for identifying severe C. pneumoniae infection was assessed using ROC curves.

**Results:**

Serum KL-6 levels were significantly higher in the severe group than in the mild group [303.00 (227.75, 328.25) U/mL vs. 146.50 (133.50, 164.25) U/mL, P < 0.001]. Binary logistic regression analysis indicated that KL-6 was independently associated with severe *C. pneumoniae* infection in children (P < 0.001), and the area under the curve (AUC) for differentiating severe infection from mild infection was 0.951 (95%CI=0.880-0.997, P<0.001). Further analysis revealed that the KL-6 level was positively correlated with the number of pulmonary lesions, the number of hospitalization days, and *C. pneumoniae*-IgM levels but not with other indicators.

**Conclusion:**

The serum KL-6 level can effectively reflect the degree of pulmonary parenchymal injury in children with *C. pneumoniae* infection and can serve as an independent biomarker for severe *C. pneumoniae* infection. It demonstrates high discriminatory ability for identifying severe cases and may serve as an auxiliary biomarker for assessing pulmonary parenchymal injury.

## 1 Introduction

*Chlamydia pneumoniae* (*C. pneumoniae*) was first isolated in 1965 from a conjunctival specimen of a child participating in a trachoma vaccine trial in Taiwan. It is among the common pathogens causing community-acquired pneumonia (CAP). *C. pneumoniae* is transmitted via respiratory droplets, and humans are the only known natural reservoir. *C. pneumoniae* infection manifests as diverse clinical phenotypes: most patients are asymptomatic or present with mild to moderate respiratory symptoms (e.g., runny nose and cough) with a long incubation period of 3–4 weeks, which is easily overlooked; a few may progress to severe pneumonia or even extrapulmonary complications. Serological tests (e.g., elevated peripheral blood IgM antibody titres) are among the gold standards for diagnosing acute *C. pneumoniae* infection (Miyashita, 2022). Clinicians often use routine inflammatory indicators such as the white blood cell (WBC) count, high-sensitivity C-reactive protein (CRP) level, and procalcitonin (PCT) level to assess disease severity, but these indicators have limited specificity for pulmonary parenchymal injury caused by *C. pneumoniae* infection (Su and He, 2025).

Krebs von den Lungen-6 (KL-6) is a large molecular glycoprotein antigen that is mainly secreted by proliferating or damaged type II alveolar epithelial cells (Gao et al., 2024). KL-6 corresponds to the shed ectodomain of mucin 1 (MUC1), a transmembrane glycoprotein constitutively expressed on the apical surface of type II alveolar epithelial cells. Basal and stress-induced MUC1 synthesis, coupled with tightly regulated ectodomain shedding, are the primary determinants of circulating KL-6 concentrations under both physiological and pathological conditions (Ballester et al., 2021; Remuzgo-Martínez et al., 2021). During pulmonary inflammation or fibrotic remodeling, matrix metalloproteinase (MMP)-mediated proteolytic cleavage of MUC1 promotes release of the soluble KL-6 ectodomain into the alveolar space, with subsequent spillover into the systemic circulation accounting for measurable elevations in serum KL-6 levels (Zamfir et al., 2023). In 1989, the Japanese scholar Kohno and colleagues first used it to evaluate the disease activity of interstitial lung disease (ILD), and in 1999, the Japanese National Health Insurance System officially included KL-6 as a biomarker for assessing ILD disease activity (Guo et al., 2024). Current detection methods for KL-6 include ELISA, chemiluminescence, and immunoturbidimetry (Wang et al., 2023). In recent years, the clinical value of KL-6 in diagnosing various lung diseases has been confirmed: it can predict the risk of severe COVID-19 pneumonia (Snopkowska Lesniak et al., 2025), is correlated with in-hospital mortality in patients with CAP (Lu et al., 2023; Heltborg et al., 2024), and can be used to monitor disease progression in patients with immune checkpoint inhibitor-induced pneumonia (Matsukane et al., 2024). However, the expression pattern of KL-6, the correlation of the KL-6 level with disease severity, and discriminatory performance of the KL-6 level for acute *C. pneumoniae* infection in children remain unclear, with few relevant studies (Achille et al., 2025).

C. *pneumoniae* infection is an important cause of CAP in children (Ma et al., 2022; Basile and Sandelich, 2025). Due to the nonspecific symptoms and insufficient sensitivity of routine inflammatory indicators for pulmonary parenchymal injury, biomarkers that can accurately reflect alveolar epithelial injury caused by *C. pneumoniae* infection are urgently needed. *C. pneumoniae* infection exacerbates alveolar epithelial injury via characteristic monocyte-macrophage-mediated inflammatory responses, thereby driving measurable elevations in circulating KL-6 levels. This study aimed to analyze the differences in serum KL-6 levels between children with mild and severe acute *C. pneumoniae* infection, explore its clinical value as an auxiliary indicator for assessing severe infection and pulmonary parenchymal injury, and provide a basis for the rational selection of clinical detection items.

## 2 Materials and methods

### 2.1 Study subjects

Children hospitalized with acute lower respiratory tract infection caused by *C. pneumoniae* at Children’s Hospital of Wujiang District, Suzhou (Wujiang Campus of Children’s Hospital Affiliated to Soochow University) from May 2024 to April 2025 were enrolled as the study group. The inclusion criteria were as follows: (1) age ≤18 years; (2) clinical diagnosis of *C. pneumoniae* infection based on the Zhu Futang practice of pediatrics (8th ed., 2015), including clinical symptoms (low-grade fever, mild respiratory symptoms such as runny nose and cough, gradually worsening cough lasting 1–2 months, and rapid breathing), laboratory diagnosis (positive for serum *C. pneumoniae*-IgM), and imaging findings (segmental pneumonia or extensive unilateral or bilateral lesions); (3) severe cases were defined as a poor general condition, refusal to eat or dehydration, consciousness disturbance, rapid respiratory rate, cyanosis, dyspnea, pulmonary infiltration involving multiple lobes or ≥2/3 of the lung, pleural effusion, pulse oxygen saturation ≤0.92, or extrapulmonary complications; and (4) no prior medication before admission, and treatment with azithromycin or erythromycin during hospitalization.

The exclusion criteria were as follows: (1) had allergies, tumors, immunodeficiency, or other congenital diseases; and (2) had mixed infections with other pathogens (*M. pneumoniae*, influenza A virus, influenza B virus, respiratory syncytial virus, human rhinovirus, or adenovirus). Healthy children who underwent a physical examination during the same period were selected as the control group. This study was approved by the Ethics Committee of our hospital (approval number: 2023023, 2025002). No additional invasive procedures were performed for research purposes, with all data fully de-identified and a waiver of informed consent granted. All methods were performed in accordance with the ethical standards of the Declaration of Helsinki.

### 2.2 Instruments and detection methods

Serum KL-6 levels were measured using a Fujifilm LUMIPULSE G1200 chemiluminescent immunoassay system (Rbio (Shanghai) Diagnostic Technology Co., Ltd.). Serum *C. pneumoniae*-IgM antibody level was measured using an iFlash 3000 chemiluminescent immunoassay system (Shenzhen YHLO Biotech Co., Ltd.). Multiplex PCR targeting Mycoplasma pneumoniae, influenza A virus, influenza B virus, respiratory syncytial virus, human rhinovirus, and adenovirus was carried out using a Gentier 96R Fully Automated Medical PCR Analysis System (Xi’an Tianlong Science and Technology Co., Ltd., Xi’an).Routine blood tests (WBC count, RBC count, PLT count, neutrophil percentage, lymphocyte percentage, neutrophil absolute value, lymphocyte absolute value, and CRP level) were performed using a Mindray BC7500 automatic hematology analyzer (Shenzhen Mindray Bio-Medical Electronics Co., Ltd.). Routine biochemical parameters (ALT, LDH, and PA levels) were analyzed using a Mindray BS-2000 automatic biochemical analyzer (Shenzhen Mindray Bio-Medical Electronics Co., Ltd.). Coagulation (D-D) was assessed using a Sysmex CS-5100 automatic coagulation analyzer (Sysmex Medical Electronics (Shanghai) Co., Ltd.), and the levels of infection markers (PCT and IL-6) were measured using a Roche e411 automatic immunoassay analyzer (Roche Diagnostics (Shanghai) Co., Ltd.),RRID: SCR_018369.Imaging examinations were performed using a GE Optima 640 whole-body CT scanner (General Electric Company, USA).

The KL-6 assay was performed using the Lumipulse^®^ G KL-6 kit, with a lower limit of detection (LoD) of 1 U/mL and a coefficient of variation (CV)≤4.4%.KL-6 was quantified using the Lumipulse^®^ G KL-6 kit (LoD: 1 U/mL; intra-assay CV ≤ 4.4%; manufacturer-recommended adult cutoff: < 500 U/mL). Multiplex PCR was performed using the Sansure Six-Pathogen Respiratory Nucleic Acid Detection Kit (intra-assay CV ≤ 5.0%) on a Gentier 96R PCR system. A result was defined as positive if typical S-shaped amplification curves were observed with Ct ≤ 40.All other routine laboratory assays were performed using original manufacturer-supplied kits. All instruments and reagents used in this study are commercially available products, not in-house research formulations.

### 2.3 Specimen detection

All the enrolled children underwent routine laboratory tests, including *C. pneumoniae*-IgM, routine blood tests, PCT, IL-6, coagulation tests, and routine biochemical tests on the day of admission, and throat swabs were collected for the nucleic acid detection of six respiratory pathogens. Children who were positive for *M. pneumoniae*, influenza A virus, influenza B virus, respiratory syncytial virus, human rhinovirus, or adenovirus were excluded. Serum samples from the remaining children who were positive for *C. pneumoniae*-IgM were collected, frozen within 1 hour after collection, and stored at -20 °C until use. After discharge, clinical data and imaging reports were reviewed to screen specimens from children who met the inclusion criteria. Serum samples stored at -20 °C were thawed at room temperature for 30 minutes, and KL-6 levels were measured using a Fujifilm LUMIPULSE G1200 chemiluminescent immunoassay system.

### 2.4 Data collection

On the first day of admission, all the children with lower respiratory tract infections underwent unified routine blood tests, measurements of biochemical parameters, and assessments of infection markers. Retrospectively collected laboratory results included the WBC count, RBC count, PLT count, neutrophil percentage, lymphocyte percentage, neutrophil count, lymphocyte count, and CRP, ALT, LDH, PA, D-D, PCT, IL-6, and *C. pneumoniae*-IgM levels. Imaging examinations were performed on the first day of admission, and the number of infected lung segments was collected retrospectively. Hospitalization days were based on discharge records.

### 2.5 Statistical analysis

GraphPad Prism 8.0 software and IBM SPSS Statistics 22 were used for all statistical analyses. Normality testing was performed for continuous variables. Normally distributed continuous data were expressed as mean ± standard deviation (x ± s) and compared using the t test. Non-normally distributed data were presented as median with interquartile range and analyzed with the Wilcoxon rank-sum test (Mann-Whitney U test). The chi-square test was used for comparisons of categorical variables. The diagnostic performance of each indicator was analyzed using ROC curves, and the AUC, optimal cut-off value, sensitivity, specificity, positive predictive value (PPV), negative predictive value (NPV), and Youden’s index were calculated. The 95% confidence interval (95% CI) of AUC was calculated using the Percentile Bootstrap method, with the number of resampling iterations set to 1000. Specifically, 1000 ROC curves were generated through repeated sampling with replacement, and the 2.5% and 97.5% quantiles of the AUC distribution were taken as the lower and upper limits of the 95% confidence interval, respectively. This method is the gold standard for confidence interval calculation in ROC analysis, which can effectively avoid the limitation of the traditional normal approximation method that the confidence interval boundaries exceed the reasonable range of 0 to 1 when the AUC is close to 1 and the sample size is relatively small. For the multivariable binary logistic regression predicting severe *C. pneumoniae* infection, a maximum of two predictor variables were entered into the model following the “10 events per variable” rule to mitigate overfitting risk. A two tailed adjusted P value<0.05 was considered to indicate statistical significance.

## 3 Results

### 3.1 Comparison of general data

A total of 68 children were included in the study group: 36 males and 32 females, with an average age of 9.71 ± 3.14 years. They were divided into a mild group (n=48) and a severe group (n=20) based on their clinical symptoms. The control group included 19 healthy children (10 males) with an average age of 8.39 ± 3.77 years. Chi-square tests for sex and t tests for age and hospitalization duration revealed no significant differences between the severe group and mild group in sex, and age (P > 0.05) (see **Figure 1** and **Supplementary Table 1**).

**Figure 1:**
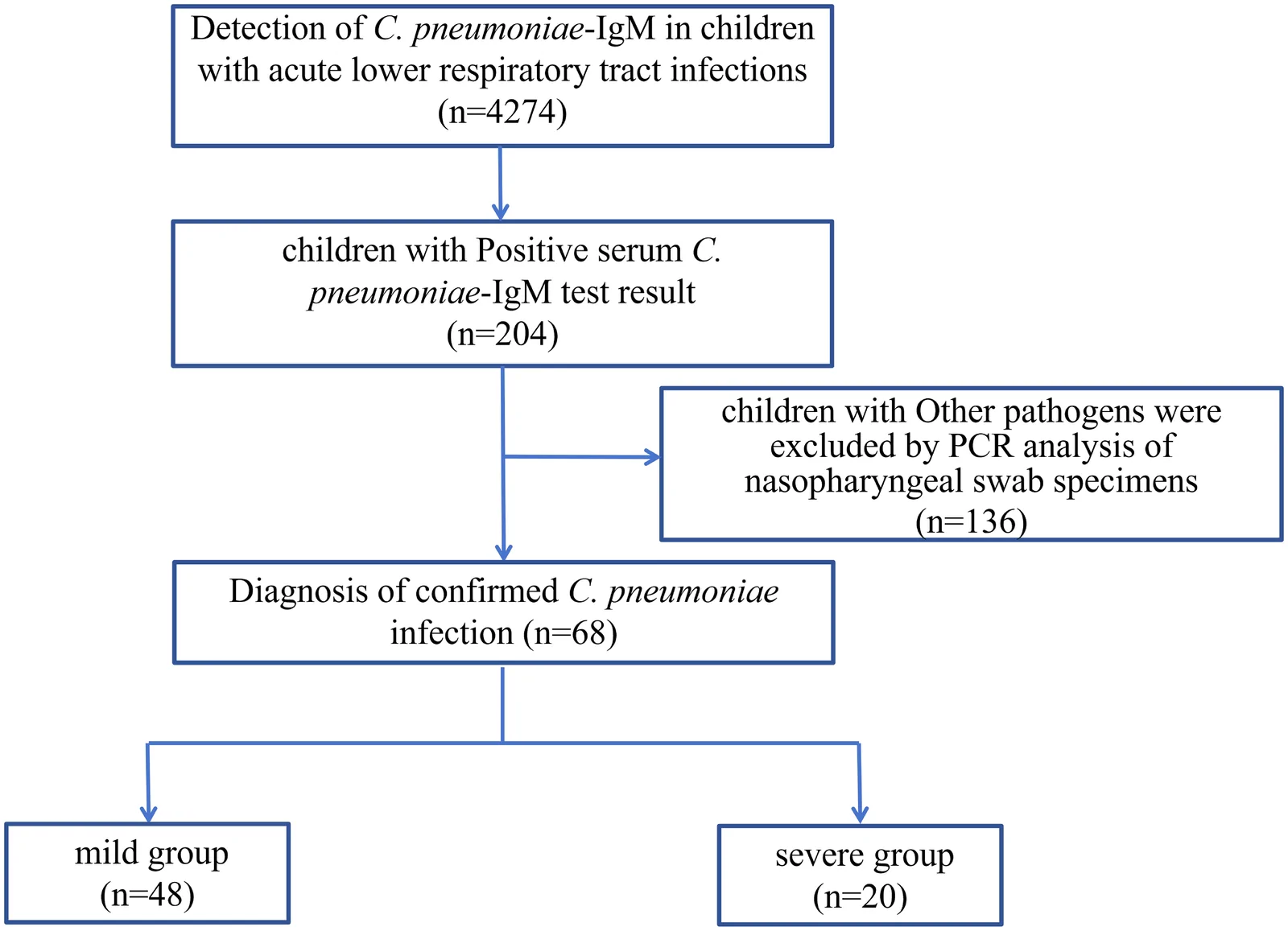
Flow chart of the study. *C. pneumoniae*.

### 3.2 Expression of KL-6 in patients with *C. pneumoniae* infection

Comparisons of laboratory data between the study group and control group revealed that the WBC count, RBC count, Neutrophil count, CRP, and KL-6 levels were significantly higher in the study group than in the control group: WBC count [9.05 (7.30, 11.98) vs. 7.65 (5.98, 8.57)], RBC count (4.86 ± 0.43 vs. 4.49 ± 0.25), Neutrophil count[5.25 (3.77, 8.00) vs. 3.00 (2.60, 5.83)], CRP level [3.70 (0.59, 15.71) vs. 0.20 (0.20, 0.95)], and KL-6 level [158.50 (142.25, 222.00) vs. 128.00 (113.00, 163.00)] (**Table 1**). Binary logistic regression analysis indicated that the RBC count (P = 0.037) and CRP level (P = 0.049) were independently associated with C. pneumoniae infection in children, while KL-6 showed a borderline association (P = 0.079) (**Figure 2A**; **Supplementary Table 2**).

**Table 1:** Comparison of basic clinical parameters between healthy controls and children with *C. pneumoniae* infection.

Variable	Control group	*C. pneumoniae* group	t/Z	P value
WBC count	7.65 (5.98, 8.57)	9.05 (7.30, 11.98)	2.230	**0.026**
RBC count	4.49 ± 0.25	4.86 ± 0.43	-4.546	**<0.001**
PLT count	288.00 (250.50, 331.50)	306.00 (259.25, 345.50)	0.659	0.510
Neutrophil percentage	52.26 ± 17.70	59.34 ± 12.82	-1.549	0.137
lymphocyte percentage	35.93 ± 18.53	31.58 ± 12.36	0.917	0.370
Neutrophil count (×10^9^/L)	3.00 (2.60, 5.83)	5.25 (3.77, 8.00)	2.769	**0.006**
lymphocyte count (×10^9^/L)	2.60 (1.84, 3.85)	2.69 (1.92, 3.53)	-0.104	0.917
PCT	0.04 (0.03, 0.04)	0.05 (0.03, 0.07)	1.323	0.186
CRP	0.20 (0.20, 0.95)	3.70 (0.59, 15.71)	3.731	**<0.001**
ALT	16.70 (11.90, 19.90)	13.50 (11.00, 17.00)	-1.707	0.088
LDH	238.23 ± 28.63	248.24 ± 57.45	-1.002	0.321
KL-6	128.00 (113.00, 163.00)	158.50 (142.25, 222.00)	3.000	**0.003**

Normally distributed continuous data were expressed as mean ± standard deviation (x ± s) and compared using the t test. Non-normally distributed data were presented as median with interquartile range and analyzed with the Mann-Whitney U test. Bold values indicate statistically significant differences between the two groups (P < 0.05).

**Figure 2:**
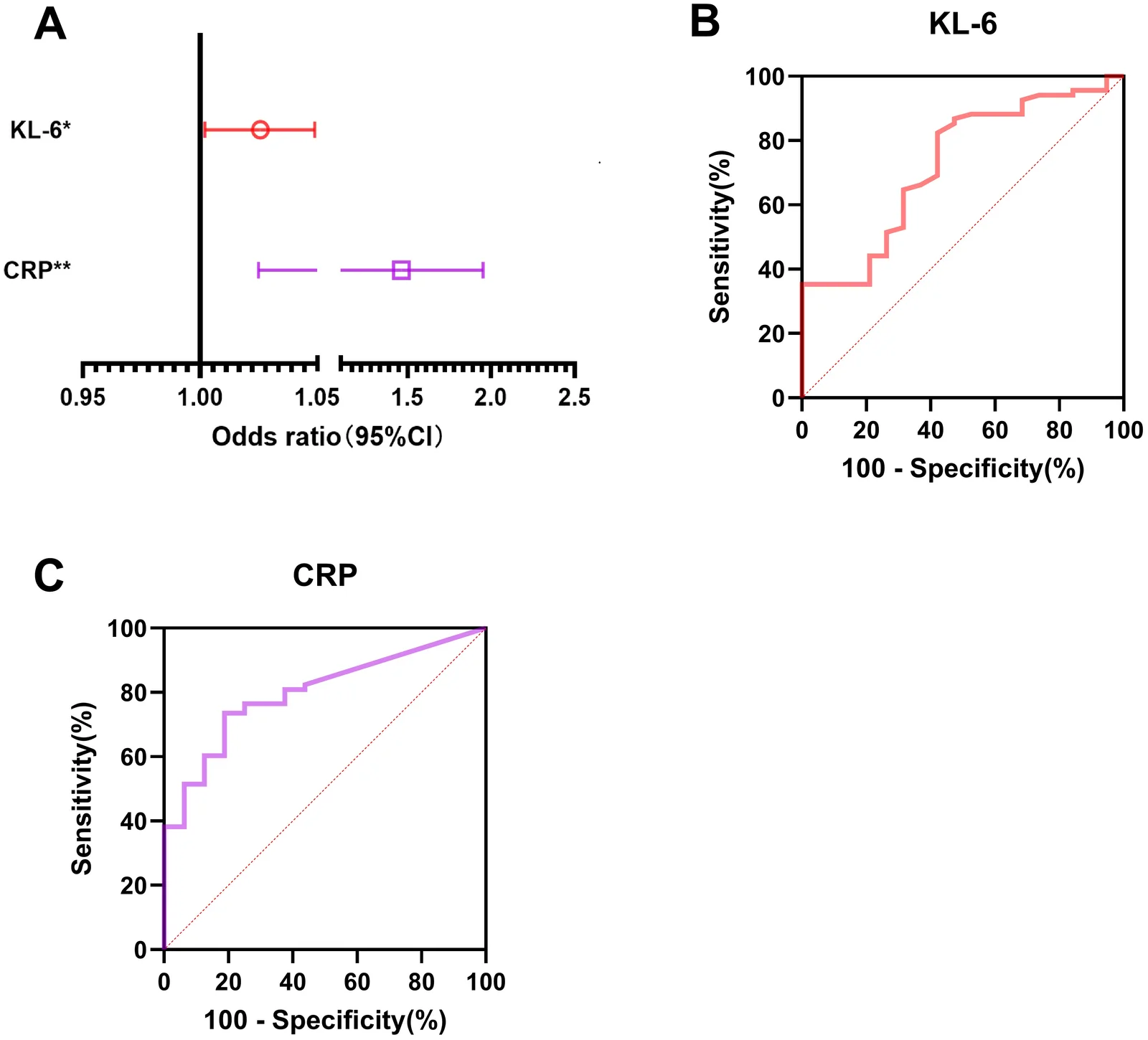
Comparative utility of the Clinical Parameters in *C. pneumoniae* infection. **(A)** The odds ratio of the Clinical Parameters in *C. pneumoniae* infection. **(B–D)**. ROC curves of KL-6, RBC, and CRP for discriminating *C. pneumoniae* infection in children from healthy pediatric controls.

The ROC curve analysis showed that the AUC values of WBC count, CRP level, and KL-6 level for distinguishing C. pneumoniae infection from healthy controls were 0.756, 0.799, and 0.759, respectively, indicating a statistically significant difference between the two groups, suggesting a potential utility in differentiating infected patients from controls (**Supplementary Table 3**).

### 3.3 KL-6 is independently associated with severe *C. pneumoniae* pneumonia in children

A comparison of laboratory data between the severe and mild groups revealed that the severe group had significantly higher KL-6 [303.00 (227.75, 328.25)] and ALT [16.10 (13.00, 19.35)] levels than the mild group [KL-6: 146.50 (133.50, 164.25); ALT: 12.00 (11.00, 16.00)]. In contrast, LDH levels were significantly lower in the severe group (230.06 ± 38.76) compared with the mild group (255.81 ± 62.44) (**Table 2**).Given that ALT and LDH both reflect hepatic function and have different scales, we standardized them into Z-scores (subtracting the mean and dividing by the standard deviation). The liver composite score was then calculated as the arithmetic mean of the two Z-scores and entered as a continuous covariate in the multivariate logistic regression model. Binary logistic regression analysis indicated that KL-6 was independently associated with severe *C. pneumoniae* infection in children (P < 0.001) (**Figure 3A**; **Supplementary Table 4**).

**Table 2:** Comparison of baseline characteristics between children with mild and severe C. pneumoniae infection.

Variable	Mild group	Severe group	t/Z	P value
*C. pneumoniae* - IgM	13.43 (4.13, 24.37)	20.15 (12.20, 29.91)	1.272	0.203
WBC count	8.60 (7.23, 11.98)	9.54 (7.53, 12.58)	0.195	0.845
RBC count	4.86 ± 0.44	4.85 ± 0.43	0.048	0.962
PLT count	298.00 (264.50, 339.50)	311.50 (254.00, 356.25)	0.458	0.647
Neutrophil percentage	59.09 ± 14.02	59.92 ± 9.64	-0.242	0.809
lymphocyte percentage	31.25 (21.85, 40.70)	26.65 (24.75, 41.68)	0.087	0.93
Neutrophil count	5.10 (3.77, 7.80)	6.29 (3.77, 8.07)	0.303	0.762
lymphocyte count	2.70 (1.91, 3.52)	2.69 (2.25, 3.68)	0.269	0.788
CRP	3.50 (1.28, 12.23)	11.53 (0.46, 22.73)	1.093	0.274
PCT	0.05 (0.03, 0.06)	0.05 (0.03, 0.07)	0.611	0.541
IL-6	8.64 (3.92, 15.07)	12.45 (2.95, 15.81)	0.076	0.94
ALT	12.00 (11.00, 16.00)	16.10 (13.00, 19.35)	2.24	**0.025**
LDH	255.81 ± 62.44	230.06 ± 38.76	2.06	**0.044**
D-D	0.28 (0.19, 0.49)	0.40 (0.23, 0.78)	1.563	0.118
KL-6	146.50 (133.50, 164.25)	303.00 (227.75, 328.25)	5.823	**<0.001**

Normally distributed continuous data were expressed as mean ± standard deviation (x ± s) and compared using the t test. Non-normally distributed data were presented as median with interquartile range and analyzed with the Mann-Whitney U test. Bold values indicate statistically significant differences between the two groups (P < 0.05).

**Figure 3:**
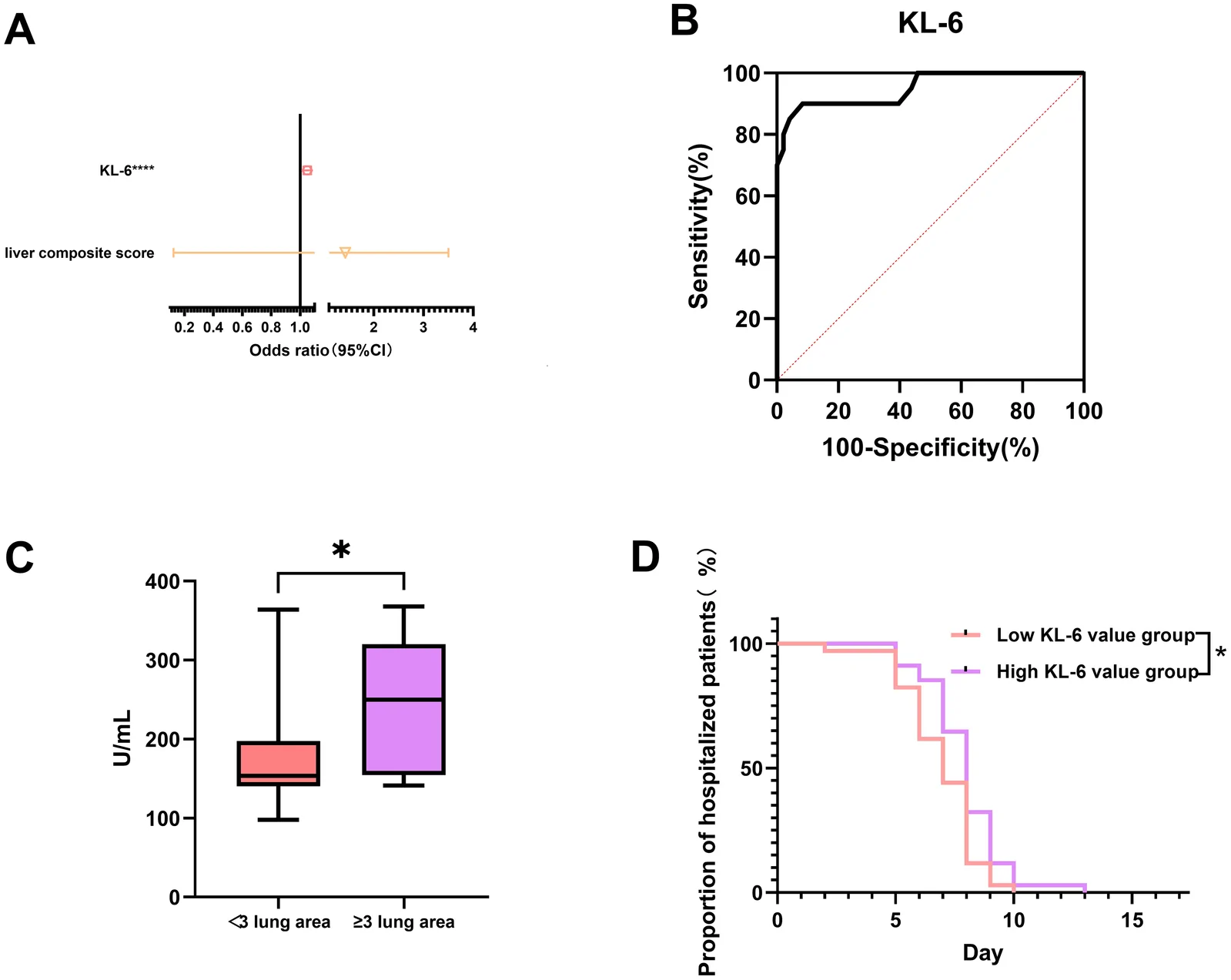
KL-6 is independently associated with severe *C. pneumoniae* infection in children. **(A)** The odds ratio of KL-6 in severe *C. pneumoniae* infection. **(B)** ROC curve of KL-6 for discriminating severe *C. pneumoniae* infection. **(C)** KL-6 level was positively correlated with the number of pulmonary lesions. **(D)** The high KL-6 level group had a longer duration of hospitalization than the low KL-6 level group. *P < 0.05.

The ROC curve analysis showed that the AUC of KL-6 levels for discriminating severe from mild C. pneumoniae infection was 0.951, indicating a statistically significant capacity to differentiate disease severity (**Figure 3B**; **Supplementary Table 5**).

### 3.4 Correlation between KL-6 levels and the severity of *C. pneumoniae* infection

To further explore the relationship between the KL-6 level and the course of *C. pneumoniae* infection, KL-6 expression analysis and imaging were performed, and the results revealed that KL-6 levels were significantly lower in patients with <3 lesions [(153.50 (140.25, 197.75)] than in those with ≥3 lesions [250.00 (154.50, 320.00)] (P = 0.011). Further analysis of patients in the high value group (n=34) and low-expression group (n=34) based on the median KL-6 level(159U/mL) revealed that the high KL-6 group exhibited longer hospitalization duration (**Figure 3D**) and higher *C. pneumoniae*-IgM levels, whereas no inter-group differences were observed for other laboratory indicators (**Figure 4**).

**Figure 4:**
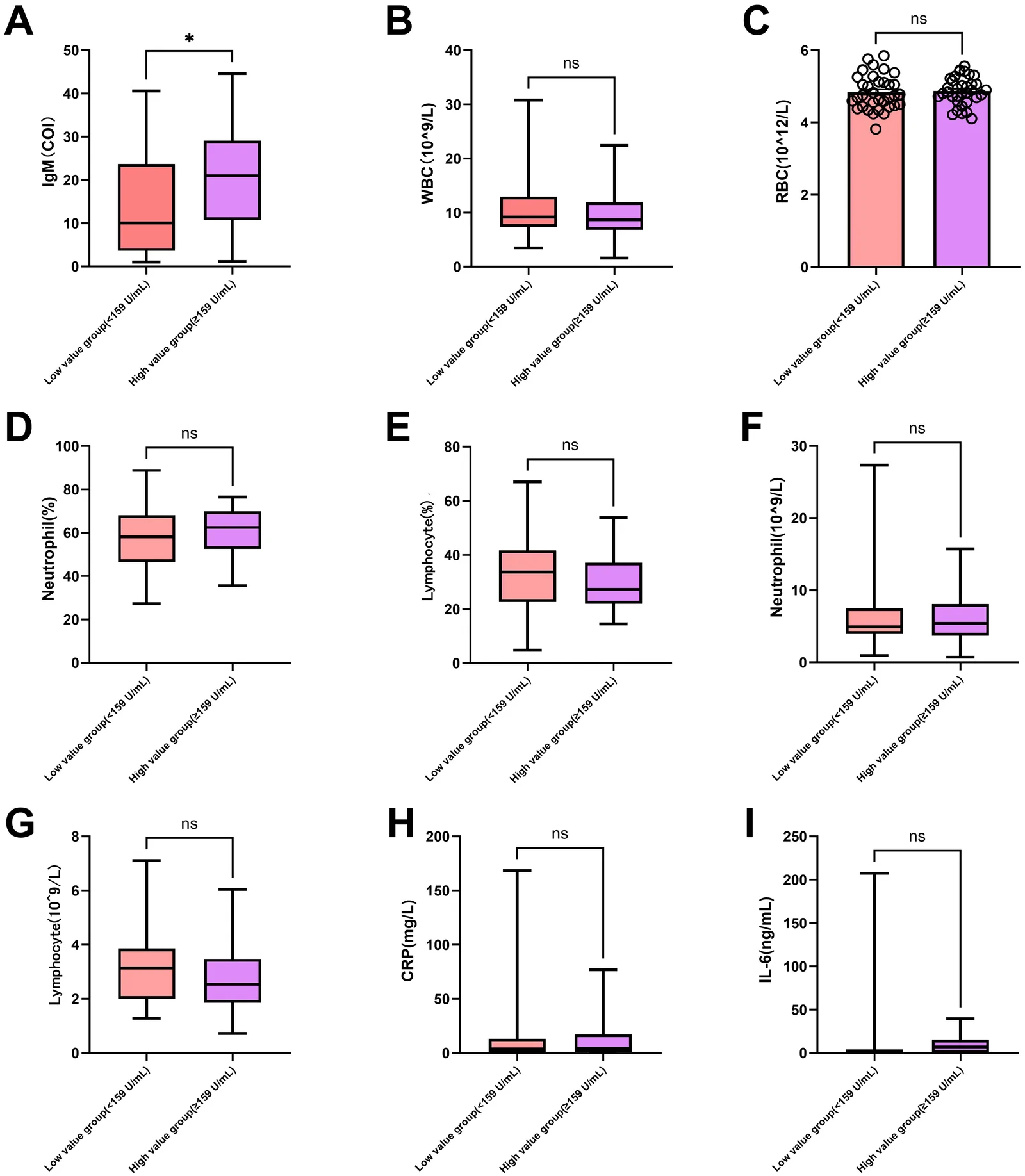
Comparison of laboratory parameters between high and low KL-6 groups in *C. pneumoniae* infection patients. Patients were divided into low value group(<159 U/mL) and high value group(≥159 U/mL) according to KL-6 levels. **(A)** Serum IgM levels; **(B)** White blood cell count (WBC); **(C)** Red blood cell count (RBC); **(D)** Neutrophil percentage; **(E)** Lymphocyte percentage; **(F)** Neutrophil count; **(G)** Lymphocyte count; **(H)** C-reactive protein (CRP); **(I)** Interleukin-6 (IL-6). Bar graphs represent the median, individual dots represent individual patient samples. *P < 0.05; ns, no significance.

## 4 Discussion

*C. pneumoniae* is an intracellular pathogen. The pathogenesis of *C. pneumoniae* infection, which involves mainly monocyte–macrophage responses, remains unclear. As carriers for the storage and transmission of pathogens, infected alveolar macrophages can migrate through the bloodstream, leading to the dissemination of Chlamydia and systemic symptoms. This mechanism is particularly crucial in *C. pneumoniae* infection. When *C. pneumoniae* infection progresses to severe cases, the extent of alveolar epithelial damage expands, potentially further exacerbating the manifestation of systemic symptoms (Tagini et al., 2025a). In recent years, the incidence of respiratory infections caused by *C. pneumoniae* has been increasing (Tagini et al., 2025b). The prevalence of *C. pneumoniae* varies among countries, ranging from 1.4% to 8.0%, and accounts for approximately 3.5% of CAP cases among Chinese children (Li et al., 2018). No significant difference in positive rates has been observed between males and females, and *C. pneumoniae* affects mainly children over 5 years old (Cai et al., 2022). In the early stage of acute *C. pneumoniae* infection, it is difficult to distinguish from infections caused by other pathogens, especially *M. pneumoniae*. After diagnosis, clinical laboratories need relevant indicators to assist in assessing disease severity, and laboratory indicators may also be required to monitor treatment effects (Miyashita, 2022). Therefore, how clinicians choose appropriate detection items is particularly important in the current context of tight medical resources.

This study is the first to confirm that the serum KL-6 level is independently associated with severe acute *C. pneumoniae* infection in children (OR = 1.048, 95% CI: 1.023–1.075, P < 0.001), with an AUC of 0.951 for discriminating severe infection. These results are consistent with the role of KL-6 in other lung diseases (Hong et al., 2024): KL-6 is mainly secreted by damaged type II alveolar epithelial cells (Ishikawa et al., 2012). When *C. pneumoniae* infection progresses to severe disease, the range of alveolar epithelial injury expands, increasing KL-6 release, and thus it can serve as a specific biomarker for pulmonary parenchymal injury. These findings are consistent with those of studies on *M. pneumoniae* pneumonia (Shu et al., 2015) and RSV bronchiolitis (Kawasaki et al., 2009), suggesting that the KL-6 level is valuable for assessing lung injury caused by different pathogens in children. However, the average KL-6 expression level in patients with *C. pneumoniae* infection is lower than that in patients with *M. pneumoniae* pneumonia, possibly because *C. pneumoniae* infection causes mainly mild to moderate lung injury, and this study excluded children with mixed infections, reducing the inflammatory effect. Prior investigations suggest a lower pediatric reference interval for KL-6 compared to adults, with Imai et al (Imai et al., 2002) reporting a 250U/mL upper limit and Kubota & Haruta (Kubota and Haruta, 2006) proposing a 300 U/mL cutoff (mean+2SD). Our moderate-severity *C. pneumoniae* cohort had a median KL-6 level of 303.00 (227.75, 328.25) U/mL, and the optimal cutoff of 196.5 U/mL fell below these proposed pediatric benchmarks.

This study also revealed that the KL-6 level increases with the number of lesions, directly confirming its effectiveness as a marker for the extent of lung injury, which is consistent with the conclusion proposed by Imai et al. that “elevated KL-6 reflects the degree of alveolar injury” (Imai et al., 2002). In addition, the KL-6 level is not significantly correlated with the levels of routine inflammatory indicators (e.g., CRP and PCT), indicating that KL-6 mainly reflects pulmonary parenchymal injury than the systemic inflammatory response, which can compensate for the deficiency of routine indicators in evaluating local lung injury.

Of note, serum LDH was marginally lower in the severe group (230.06 ± 38.76 U/L) compared with the mild group (255.81 ± 62.44 U/L, P = 0.044).Mean LDH concentrations for both severe and mild patients fell within our healthy pediatric reference range (238.23 ± 28.63 U/L; infected vs control group, P = 0.321). The inter-group LDH difference was smaller than intrinsic biological variation among healthy children, suggesting LDH was not systemically activated in this C. pneumoniae cohort. This borderline statistical result is partly attributable to the small sample size of severe cases (n = 20) and retrospective sampling on admission. These findings fit the pathogenic features of C. pneumoniae. This obligate intracellular pathogen triggers chronic, monocyte-macrophage-predominant non-necrotic inflammation localized mainly to airway mucosa, without widespread acute pulmonary parenchymal necrosis or membrane injury, thereby limiting systemic LDH release. Multiple pediatric cohorts corroborate this view: most inflammatory markers were normal in 145 children with C. pneumoniae pneumonia (Wang et al., 2026); in a 141-patient risk model, only wheezing (OR = 15.47) and co-infection (OR = 8.25) were independent predictors of severe disease, whereas necrosis-associated markers such as LDH were excluded (Li et al., 2026). LDH therefore is unsuitable for severity assessment in isolated C. pneumoniae infection. The limitations of routine inflammatory markers further highlight the complementary diagnostic value of lung-specific KL-6 for recognition of severe infection.

This study revealed that KL-6 is a serum marker of the severity of *C. pneumoniae* infections. If KL-6 levels continue to increase, a severe infection should be suspected, and timely intervention should be implemented to identify serious risks early. KL-6 can be used as a supplementary detection indicator for suspected severe *C. pneumoniae* cases, especially when routine inflammatory indicators are normal but clinical symptoms progress, helping to detect pulmonary parenchymal injury early. Given that *C. pneumoniae* infection is mainly mild, KL-6 does not need to be a routine screening indicator, but for children with severe symptoms such as persistent cough and rapid breathing, KL-6 detection can improve the accuracy of disease assessment and optimize the allocation of medical resources.

This study has certain limitations. It was a single-center small-sample study with only 20 severe cases among 68 enrolled children, which may have affected the statistical power. Changes in KL-6 levels before and after treatment were not tracked, and thus the value of KL-6 levels in monitoring treatment effects could not be evaluated. Children with mixed infections or underlying diseases were excluded; therefore, the results may not be generalizable to patients with complex cases. Experiments to determine the molecular mechanism of action of KL-6 in alveolar epithelial cell injury were not performed; Our findings can only establish associations between KL-6 levels and disease severity, rather than causal relationships. Future work can further optimize the translational utility of KL-6 in pediatric *C. pneumoniae* infection management through multicenter prospective investigations, longitudinal serial monitoring of KL-6 dynamics, inclusion of patients with complex comorbidities or mixed infections, and rigorous mechanistic dissection of MUC1-mediated KL-6 release pathways.

## 5 Conclusions

The serum KL-6 level serves as a valuable biomarker for assessing the severity of C. pneumoniae infection in children, exhibiting strong discriminatory performance for distinguishing severe from mild disease (AUC = 0.951). It can be used as an auxiliary indicator for evaluating pulmonary parenchymal injury in suspected cases, providing a basis for the precise management of *C. pneumoniae* infection.

## Data Availability

The raw data supporting the conclusions of this article will be made available by the authors, without undue reservation.
